# DIGITAL HEALTH AND OLDER ADULT WELL-BEING: THE MEDIATING ROLE OF SOCIAL TECHNOLOGY USE

**DOI:** 10.1093/geroni/igae098.3836

**Published:** 2024-12-31

**Authors:** Shannon Power

**Affiliations:** University of Georgia, Athens, Georgia, United States

## Abstract

Older adults engage in digital health use (DHU) and social technology use (STU) to connect with health care providers and loved ones. DHU has expanded as an accessible health care option, though its effects on well-being are underexplored. Meanwhile, STU is known to improve quality of life for older adults. This study investigates the association between DHU and well-being and determines whether STU mediates this association. Data from the 2022 National Health and Aging Trends Study were analyzed, with a sample of 5,773 U.S. Medicare beneficiaries, ages 65 and older. The sample was primarily White (64%), female (58%), 65-79-years-old (52%), and urban-living (84%). Structural equation modeling captured the complex relationships of the latent constructs of DHU (e.g., telehealth visit, sending portal messages), STU (e.g., video chat, texting, social media), and well-being (e.g., belief in one’s life purpose, self-determination, confidence). Confirmatory factor analyses with sufficient fit indices established validity of the measurement models. In the full model, DHU was negatively associated with well-being (β=-0.22, p<.001); however, a mediation analysis revealed a strong partial mediation (β=0.32 (CI 0.24-0.46), SE=0.055, z=6.163, p<.001) with acceptable model fit. Adding STU as a mediator reversed the direction and increased the magnitude of the association between DHU and well-being. The significant negative path between DHU and well-being may indicate that DHU is not a preferred option for older adults, but the partial mediation suggests that STU offers familiarity with communication technology that strengthens the association towards well-being. Future research should clarify the reasons underlying these significant associations.

